# Dysfunctional endothelial‐derived microparticles promote inflammatory macrophage formation via NF‐кB and IL‐1β signal pathways

**DOI:** 10.1111/jcmm.13950

**Published:** 2018-10-18

**Authors:** Yanfang Wang, Jie Liu, Xiaoli Chen, Huimin Sun, Sheng Peng, Yashu Kuang, Jingjiang Pi, Tao Zhuang, Lin Zhang, Zuoren Yu, Brain Tomlinson, Paul Chan, Yihan Chen, Yuzhen Zhang, Ying Li

**Affiliations:** ^1^ Key Laboratory of Arrhythmias of the Ministry of Education of China Research Center for Translational Medicine Shanghai East Hospital Tongji University School of Medicine Shanghai China; ^2^ Department of Medicine and Therapeutics The Chinese University of Hong Kong Hong Kong SAR China; ^3^ Division of Cardiology Department of Internal Medicine Wan Fang Hospital Taipei Medical University Taipei Taiwan

**Keywords:** acute coronary syndrome, endothelial microparticles, interleukin‐1beta, NF‐kappa B, vascular inflammation

## Abstract

**Background:**

Circulating endothelial‐derived microparticles (EMPs) are reported to be increased in acute coronary syndrome (ACS). However, it remains unclear whether EMPs from dysfunctional endothelium participate in the initiation and progression of ACS and what the underlying mechanisms might be.

**Methods:**

Plasma EMPs were measured in 22 patients with ACS and 20 control patients without coronary artery diseases. EMPs from dysfunctional human umbilical vein endothelial cells (HUVECs) stressed by serum‐starvation or hypoxia were compared to the EMPs from healthy HUVECs. Confocal and fluorescent microscopy was used to visualize the incorporation of EMPs into monocytes and the translocation of NF‐кB. Monocyte adhesion, cell proliferation, and phagocytosis were detected by PKH26 red fluorescent labelling, Ki67 immunostaining, and Sudan IV staining for uptake of oxidized low‐density lipoprotein, respectively.

**Results:**

Plasma EMPs was significantly increased in ACS patients compared to controls. EMPs were incorporated into monocytes and EMPs from stressed HUVECs produced more pro‐inflammatory cytokines compared to vehicle control, which was depended on NF‐кB and IL‐1β signal pathways. EMPs from dysfunctional endothelium promoted monocyte adherence via NF‐кB and IL‐1β‐mediated MCP‐1 and CCR‐5 signals, as well as proliferation via the NF‐кB and IL‐1β‐mediated Cyclin D1 signals. Finally, EMPs from dysfunctional endothelium showed greater promotion of macrophage phagocytosis forming foam cells to produce more pro‐inflammatory cytokines.

**Conclusion:**

MPs might be involved in the inflammatory process in patients with ACS via NF‐κB and IL‐1β‐dependent signals. Targeting EMP‐mediated inflammatory responses may be a promising therapeutic strategy to limit the progression of disease in ACS.

## 1 INTRODUCTION

Atherosclerosis has been recognized as a chronic inflammatory disease. Dysfunctional endothelial cells (ECs) promote leukocyte adhesion and migration, which is a critical step in the initiation and progression of atherosclerotic lesion development leading to coronary artery diseases.^1^, ^2^, ^3^ In acute coronary syndrome (ACS), circulating monocytes were shown to express high plasma levels of monocyte chemoattractant protein‐1 (MCP‐1), which is the most important chemokine that regulates migration and infiltration of monocytes/macrophages.^4^ Toll‐like receptors (TLRs) were shown to be especially important for monocyte activation and the stimulation of TLRs activated the pro‐inflammatory transcription factor nuclear factor κB (NF‐κB), resulting in the production of cytokines that augmented local inflammation.^5^

Microparticles (MPs), the sub‐cellular particles of a size <1 μm, are derived from the plasma membrane of many eukaryotic cells in response to various biological processes such as cellular activation and apoptosis. It was reported that MPs were also present in excess numbers in disease states such as ACS,^6^, ^7^ and that the circulating MP levels were correlated with the clinical outcomes, markers of myocardial damage, inflammation, and indices of microvascular dysfunction.^8^, ^9^, ^10^

EMPs have recently emerged as a surrogate marker of endothelial injury and cardiovascular risk, and high circulating EMP levels were observed in patients with ACS.^11^, ^12^, ^13^ EMPs are reported to indicate the status of endothelial dysfunction in ACS and independently predict future cardiovascular events when combined in a multiple biomarker strategy, including high‐sensitivity C‐reactive protein (hs‐CRP).^14^ Locally produced MPs are potent pro‐inflammatory particles contributing to the local coronary inflammatory processes in patients with ACS or within the occluded coronary artery in patients with ST segment elevation myocardial infarction (STEMI).^14^, ^15^, ^16^, ^17^

Although many studies confirmed the ability of EMPs to promote vascular inflammation,^7^, ^15^, ^16^, ^17^, ^18^ few studies clarified the mechanisms of EMP‐dependent inflammatory responses contributing to cardiovascular disease progression. Therefore, the aim of this study was to explore the mechanisms by which EMPs influenced vascular inflammation via modulating macrophages infiltration and function, thereby providing evidences for a mechanism by which EMPs might promote vascular in patients with ACS, and these findings represented the novelty of the study.

## 2 MATERIAL AND METHODS

### 2.1 Patients and control subjects

A case‐control study was conducted to investigate the levels of EMPs in ACS, and 22 ACS patients complaining of chest pain in the internal medicine cardiovascular ward of Shanghai East Hospital were recruited, with 20 hospitalized patients with normal computer tomography coronary angiography acting as controls. The diagnosis of ACS was based on the American Heart Association criteria.^3^ Blood samples were drawn in the ward within 6 hours of making the diagnosis of ACS.

The institutional review board of Tongji Medical School affiliated Shanghai East Hospital approved the study protocol, all studies were performed in accordance with relevant guidelines and regulations, and written informed consent was obtained in all cases.

### 2.2 Flow cytometry assessment and quantification for patient plasma EMPs

The EMPs was measured by flow cytometry as previously described.^19^, ^20^, ^21^ In brief, patient blood samples were collected in citrated tubes and platelet‐poor plasma (PPP) obtained after centrifugation for 6 minutes at 1000 *g*. The resuspended PPP 50 μL was incubated with 5 μL fluorescein isothiocyanate (FITC)‐labelled anti‐Annexin V (556420, BD pharmingen, USA) or 10 μL of phycoerythrin (PE)‐labelled anti‐CD144 (VE‐cadherin, 561714, BD pharmingen, USA) for 20 minutes with gentle orbital shaking, then 50 μL standard MPs (Beckman Coulter) added followed by 1 mL phosphate buffered saline (PBS). The MP samples present in PBS were analyzed regarding size and fluorescence by flow cytometry in FACS Arial I (BD Bioscience, USA). On a log forward scatter/log side scatter dot plot, we defined the upper size limit of the MPs using 1 μm calibrant beads and drew a gate around the MP population. Only the MPs included in this gate were further analyzed for their fluorescence, and 5000 standard beads were counted and used to calculate the concentrations of EMPs for each sample. EMPs were defined as elements with a size <1 μm that were positively labelled with FITC‐annexin V and PE‐CD144.

### 2.3 Patient plasma protein array

Serum samples from three ACS and three non‐ACS patients were collected and total protein concentrations were quantified by BCA protein Assay Kit (KC430, Kangchen, Bio‐Tech). The samples were performed for RayBio® C‐Series Human Atherosclerosis Antibody Array C1 (AAH‐ATH‐1‐8, RayBiotech) of KangChen Bio‐Tech, Shanghai, China, according to the manufacturer's manual by blocking, incubation and detection. Data from these arrays were analyzed (http://www.kangchen.com.cn/ support/Supportmain.asp?ID=129). Twofold and greater changes were considered significant. Hierarchical clustering was used to generate heatmap in order to improve the understanding of the systematic variations in pro‐inflammatory cytokines levels between ACS and non‐ACS patients.

### 2.4 Cell culture

Human umbilical vein endothelial cells (HUVECs, PCS‐100‐013, ATCC USA) were cultured in EC growth media with SupplementMix (PromoCell, Germany) at 37°C, 5% of CO_2_ and cells of passage 4‐8 were used. The human monocytes THP‐1 (TIB‐202, ATCC USA) were cultured in RMPI 1640 culture medium (Gibco, USA) supplemented with 10% of FBS and the cell density was kept between 1 × 10^6^ and 5 × 10^6^ cells in a 25 cm^2^ flask.

### 2.5 Generation and quantification of EMPs from HUVECs

EMPs were generated from HUVECs as previously described.^20^, ^21^, ^22^ In brief, confluent cells were starved by culture in basal media without growth media supplements,^20^ or subjected to hypoxia,^22^ to induce apoptosis. The supernatant of serum‐starved or hypoxia exposed HUVECs was collected and centrifuged at 1500 *g* for 15 minutes to remove cell debris. Then the supernatant was further centrifuged at 20 000 *g* for 40 minutes and the EMPs collected for further characterization. A 50 μL of EMPs were mixed with 5 μL of FITC‐labelled anti‐Annexin V and 10 μL of PE‐labelled anti‐CD144. After incubation at room temperature for 20 minutes, the labelled EMPs were washed two times and resuspended in 100 μL PBS for flow cytometry according to the method described in quantification of patient EMPs.

HUVECs cultured under normal condition rarely produce EMPs. HUVECs cultured with starvation or under hypoxia condition produce significantly increased EMPs compared to the HUVECs under normal culture condition, similar to the significantly increased circulatory EMP levels in ACS patients compared to non‐ACS patients.

For functional study of the EMPs, the supernatant from serum‐starved or hypoxia‐exposed HUVECs cultured in a 10 cm dish was collected and centrifuged at 1500 *g* for 15 minutes to remove cell debris. Then, centrifuged at 20 000 *g* for 40 minutes and the EMPs collected, which were resuspended with 100 μL PBS and added to the THP‐1 culture in 1:50 dilution. The same procedure of EMPs preparation was performed in HUVECs under normal culture conditions which were used as vehicle control to compare with EMPs from HUVECs with starvation and under hypoxia condition, which were considered as EMPs from dysfunctional ECs.

### 2.6 EMPs incorporation to THP‐1 monocyte membrane by confocal microscopy

To visualize the incorporation of EMPs on monocyte/macrophages in a time‐dependent manner, EMP‐derived from HUVECs were labelled with PKH26 red fluorescent cell liner Mini Kit (Mini26‐1KT, Sigma‐Aldrich, USA) for 30 minutes, washed twice, and resuspended in PBS, and these red fluorescence‐labelled EMPs were added to the THP‐1 culture medium. Images were taken at 2 and 4 hours by a confocal microscopy (Leica TCS SP5 AOBS, Germany) to visualize the incorporation of EMPs into the membrane of THP‐1 monocytes. Further studies were performed using the same amount of empty PKH26 red fluorescent dye but without EMPs as a control for dye‐labelled EMPs derived from HUVECs to rule out the non‐specific lipophilic binding of non‐incorporated dye to the membrane of monocyte/macrophage (Supplemental Figure S4A).

### 2.7 Real time quantitative PCR assay

Total RNA from cells was extracted with Trizol™ Reagent (Invitrogen, Carlsbad, CA, USA). Reverse transcription was carried out by a TaKaRa Primescript™ RT reagent kit (TaKaRa, Japan) according to the manufacturer's instructions. The quantification of gene expression was performed by real time quantitative PCR on a QuantStudio™ 6 Flex Real‐time PCR system (Applied Biosystems, USA). The Ct values were normalized for the housekeeping gene GAPDH, and then the relative gene expression was calculated using expression of vehicle treated cells as a control. Primers used for quantitative real time PCR were included in Supplementary Table S1.

### 2.8 Western blotting and ELISA assay

THP‐1 cells pellets were extracted in RIPA lysis buffer (Beyotime, China) containing protease inhibitor cocktail (Calbiochem, USA). Protein concentrations were determined by a BCA kit (Pierce, USA). Equal amounts of cell extracts were electrophoresed on sodium dodecyl sulfate‐polyacrylamide gels, and then transferred onto polyvinylidene difluoride membranes (Millipore, USA). The membranes were blocked in 5% non‐fat dried milk in TBS‐T at room temperature for 2 hours and incubated with indicated primary antibodies at 4°C overnight. The primary antibodies used in the study were: anti phospho‐NF‐кB (Ser468) (1:1000, #3039, Cell signaling technology, USA), anti‐TNFα (1:1000, MAB610, R&D systems, USA), and anti β‐actin (1:1000, #3700, Cell signaling technology, USA). The membranes were washed three times with TBS‐T for 10 min each time, and then incubated with IRDye‐680LT Goat Anti‐Mouse IgG (H+L) (Li‐Cor, USA) or IRDye‐800CW Goat Anti‐Rabbit IgG (H+L) (Li‐Cor, USA) (1: 10,000 in TBS‐T) at room temperature for 1 hour. After washing in TBS‐T for another three times, the membranes were analyzed by the Odyssey infrared imaging system (Li‐Cor, USA).

Levels of IL‐1β were determined in EC supernatants by an ELISA assay kit (DLB50, R&D Systems, USA) according to the manufacturer's instruction.

### 2.9 Immunostaining

THP‐1 Cells cultured on sterile glass cover slips in 24‐well plates were fixed with 4% of paraformaldehyde (PFA) in PBS, washed, and permeabilized with 0.1% of Triton X‐100 PBS. The cells were blocked with 1% goat serum for 1 hour and incubated with primary antibodies: rabbit anti‐NF‐кB (1:1000, #8424, Cell signaling technology, USA) or rabbit anti‐Ki67 (1:500, ab16667, Abcam). After washing, the cells were incubated with Alexa Fluor 488‐conjugated donkey anti‐rabbit secondary antibodies (1:500, A21206, Invitrogen, USA) for 45 min. Then, the cover slips were mounted with Prolong Gold Anti‐fade reagent with 4′,6‐diamidino‐2‐phenylindole (DAPI) (P36935, Invitrogen, USA). The immunostaining images were captured under a Leica fluorescent microscopy (DM6000B, Leica, Germany).

Monocyte/macrophages are phagocytic cells, which engulf oxidized low‐density lipoprotein (ox‐LDL) to form foam cells and produce pro‐inflammatory cytokines. Actin cytoskeletal remodelling is required for the process of phagocytosis of monocyte/macrophages, thus actin immunostaining was performed using Alexa Fluor 488 conjugated‐phalloidin (1 mg/mL for 15 minutes, A12379, Invitrogen, USA) and quantification by calculating the relative cell area as the actin filament cytoskeletal spreading, which reflected the capacity of phagocytosis as previous described.^23^

### 2.10 Uptake of oxidized low‐density lipoprotein by Sudan IV staining for macrophage phagocytosis activity

Oxidized low‐density lipoprotein (ox‐LDL) uptake by THP‐1 monocytes was used to determine the phagocytosis activity as previously described.^24^ THP‐1 cells (5 × 10^5^) were plated in 24‐well plates and treated with vehicle control and EMPs, as well as EMPs with NF‐кB inhibitor ammonium pyroolidinedithiocarbamate (PDTC) and IL‐1β inhibitor diacerein for 48 hours, and then ox‐LDL (50 μg/mL, Yiyuan Biotechnologies, China) was incubated with these cells for 9 hours. After being fixed with 4% PFA for 10 min, the cells were stained with Sudan IV for 6 min, and rinsed with 80% ethanol 3 times. The cell images were captured under a Leica fluorescent microscopy and the percentage of the Sudan IV positive cells of the total cells was used for quantification of macrophage phagocytosis activity.

### 2.11 Monocyte adhesion assay

Adhesion of monocytes to ECs was performed as previously described.^25^ In brief, 5 × 10^4^ THP‐1 cells were treated with dysfunctional EMPs or vehicle control for 48 hours, then labelled with 5 mM PKH26 red fluorescence cell linker Mini kit (Mini26‐1KT, Sigma‐Aldrich, USA) and added into 70%‐80% confluent HUVECs in a 96‐well plate. The non‐adherent cells were removed by rinsing with PBS three times after incubation at 37°C for 1 hour. Monocyte‐endothelial adhesion was quantified by measuring the fluorescence at excitation and emission wave lengths of 485 and 535 nm, respectively, and counting the number of attached monocytes in 10 random high‐power (400×) microscopic fields of images taken by Leica fluorescence microscopy. Wells containing ECs without monocytes served as blank control. To investigate whether the adhesion is dependent on the NF‐кB or IL‐1β pathway, NF‐кB, or IL‐1β specific inhibitors PDTC (1 μM) and diacerein (50 μM) were added to see whether PDTC or diacerein reversed the EMP‐induced increased monocyte adhesion to ECs.

### 2.12 Monocyte proliferation assay

Ki67 immunostaining and cell count assay were used to evaluate the cell proliferation. In the cell count assay, 1 × 10^5^ THP‐1 cells per well were seeded in 12‐well plates and treated with EMPs. The cells of each well were harvested at 24, 48, 72 hours and the number of trypan blue negative live cells were counted under a hemocytometer. PDTC (1 μM) and diacerein (50 μM) were added to test whether monocyte/macrophage proliferation is dependent on the NF‐кB and IL‐1β pathways.

### 2.13 Reagents

Unless indicated, all chemicals and solvents were from Sigma‐Aldrich. NF‐кB inhibitor ammonium pyroolidinedithiocarbamate (PDTC) (S1808, Beyotime Biotechnology, China) was dissolved in PBS at 1 mM stock solution and used at a final concentration of 1 μM, IL‐1β inhibitor diacerein (D9302, Sigma‐Aldrich, USA) was dissolved in DMSO at 2.5 mM stock solution and used at a final concentration of 50 μM. Ox‐LDL (YB‐002, Yiyuan Biotechnologies, China) was used at a final concentration at 50 μg/mL. All stock solutions were protected from light and kept at −20°C, except ox‐LDL which was stored at 4°C. The solvent was used as a control vehicle in all experiments.

### 2.14 Statistical analysis

Data were presented as mean ± SEM of the indicated number of independent experiments. All data were analyzed by one‐way analysis of variance (ANOVA), and the least significant difference (LSD) post‐hoc test was used for multiple comparisons. Statistical analyses were performed using the SPSS software version 19.0 (SPSS Inc., USA). *P* < 0.05 was considered statistically significant.

## 3 RESULTS

### 3.1 Basic clinical characteristics of patients

Basic clinical characteristics of patients were shown on Table 1. There were no significant differences between ACS and non‐ACS patients with respect to age, sex, blood pressure, fasting serum glucose, and blood lipid levels (*P* > 0.05). In contrast, the high‐sensitivity troponin T (hs‐TNT, *P* = 0.021) and CK‐MB (*P* = 0.045) in ACS patients was significantly higher than that in non‐ACS patients, and ACS patients had significantly higher hs‐CRP level (*P* = 0.018) which indicates higher inflammatory status.

**Table 1:** Clinical characteristics of patients with ACS and non‐ACS patients

	Non‐ACS	ACS	*P* value
	Mean (95% CI)	Mean (95% CI)	
Number	20	22	
Male, %	65.0	72.7	0.325
Age, years	62.9 (56.6‐68.8)	67.7 (63.4‐72.1)	0.133
hsCRP, mg/mL	1.59 (0.91‐2.27)	3.62 (2.31‐4.94)	0.018
SBP, mmHg	123 (118‐129)	132 (123‐142)	0.078
DBP, mmHg	74.1 (67.4‐80.8)	73.9 (67.0‐80.8)	0.967
TC, mmol/L	4.10 (3.44‐4.74)	4.37 (3.79‐4.96)	0.514
LDL‐C, mmol/L	2.65 (2.05‐3.22)	2.75 (2.33‐3.18)	0.675
HDL‐C, mmol/L	1.13 (0.99‐1.26)	1.09 (1.00‐1.38)	0.393
TG, mmol/L	1.38 (1.09‐1.66)	1.39 (0.88‐1.99)	0.926
Lp(a), mg/L	71.7 (19.8‐163)	306.6 (55.4‐557)	0.062
Fasting glucose, mmol/l	5.30 (4.49‐6.11)	7.01 (5.67‐6.81)	0.154
HbA1C, %	6.10 (5.55‐6.71)	7.05 (6.30‐7.79)	0.073
Creatinine, μmol/L	76.7 (63.5‐89.8)	83.3 (75.4‐92.0)	0.312
Uric acid,	394 (313‐475)	336 (298‐373)	0.153
CK‐MB, ng/mL	3.88 (1.40‐12.00)	10.88 (5.47‐16.29)	0.041
hs‐TNT, ng/mL	0.010 (0.003‐0.038)	0.254 (0.088‐0.421)	0.015

Values are given as mean and 95% confidence interval.

ACS, acute coronary syndrome; hs‐CRP, high‐sensitivity C‐reactive protein; SBP, systolic blood pressure; DBP, diastolic blood pressure; TC, total cholesterol; LDL‐C, low density lipoprotein cholesterol; HDL‐C, high density lipoproteincholesterol; TG, triglyceride; CK‐MB, creatine kinase‐MB; hs‐TNT, high sensitivity troponin T.

### 3.2 Elevated circulating EMPs with increased inflammatory status in ACS patients

**Figure 1:**
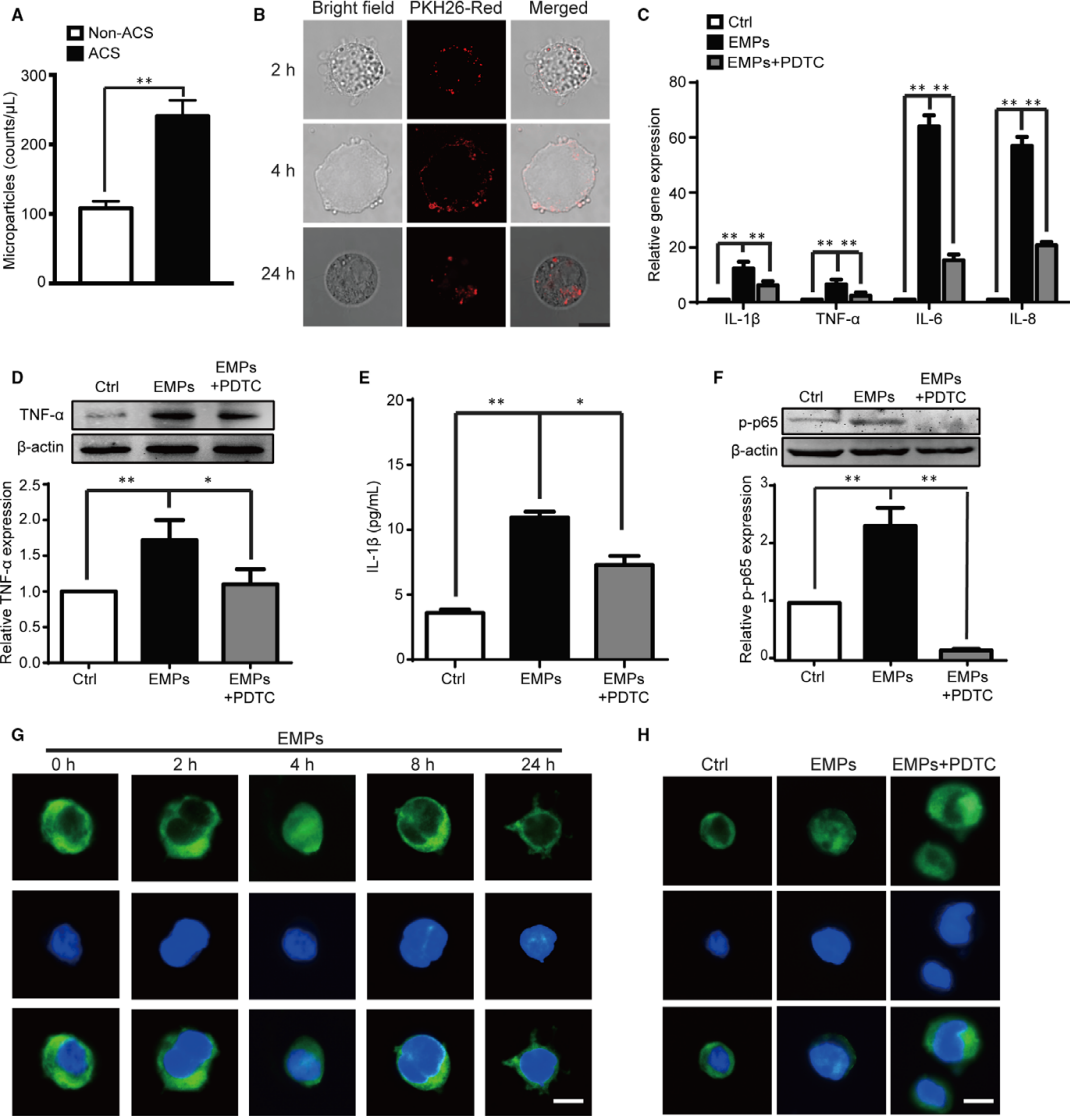
Increased circulatory EMP levels in ACS patients and dysfunctional HUVEC‐derived EMPs promote monocyte activation through NF‐кB signal pathway. A, Significantly increased circulatory EMP levels are observed in ACS patients compared to non‐ACS patients. B, Confocal microscopy images show that EMPs from serum starved HUVECs labelled with PKH26 red fluorescence are incorporated to the membrane of THP‐1 cells at 2 and 4 hours and internalized into the cells at 24 hours. Scale bar: 10 μm. C, Quantitative PCR analysis shows elevation of pro‐inflammatory cytokines (IL‐1 β, TNF‐α, IL‐6, or IL‐8) in THP‐1 cells following treatment of the EMPs from dysfunctional ECs for 24 hours and PDTC (1 μM) reversed the EMP‐induced elevation of these pro‐inflammatory cytokines. D, Western blot shows elevation of TNF‐α protein expression in THP‐1 cells following treatment of the EMPs from dysfunctional ECs for 24 hours and PDTC (1 μM) reversed the EMP‐induced elevation of TNF‐α protein. E, ELISA analysis of THP‐1 cell supernatants shows elevated IL‐1β secretion following treatment of EMPs from dysfunctional ECs for 24 hours and PDTC (1 μM) reversed the EMP‐induced secretion of IL‐1β. F, Western blot shows increased NF‐кB (p65 subunit) phosphorylation in THP‐1 cells after treatment with EMPs for 4 hours, and the NF‐кB specific inhibitor PDTC (1 μM) reversed the enhanced EMP‐induced NF‐кB p65 subunit phosphorylation with quantification shown in bottom panel. G‐H, The representative image of immunofluorescent staining shows elevated NF‐кB (p65 subunit) nuclear localization after treatment with the EMPs for 4 hours and back to the cytoplasm after 8 hours (G), and PDTC (1 μM) inhibits the EMP‐induced p65 subunit nuclear translocation at 4 hours (H). Scale bar: 10 μm. All data are presented as mean ± SEM (n = 5), *P < 0.05 and **P < 0.01

The plasma EMPs were determined by flow cytometry using CD144 and Annexin V antibody and the double positive MPs with diameters under 1.0 μm considered as EMPs (Supplemental Figure S1). The results showed that ACS patients had significantly higher plasma EMP levels compared to the non‐ACS patients (Figure 1A, *P* < 0.001).

An exploratory plasma protein array heatmap analysis in three patients with ACS and three non‐ACS patients showed that ACS patients had higher plasma levels of pro‐inflammatory cytokines TNF‐α and IL‐1β compared to non‐ACS patients (Supplementary Figure S2), providing supporting evidence for inflammation in ACS patients.

### 3.3 EMPs are incorporated into monocytes and EMPs from dysfunctional ECs produce more pro‐inflammatory cytokines via NF‐кB pathway

As describe above, ACS patients had higher circulatory EMP levels compatible with vascular inflammation. Therefore, we hypothesized that the EMPs from dysfunctional ECs that may be present in ACS patients might influence monocytes and produce more pro‐inflammatory cytokines. In order to study the mechanisms of the effects of EMPs, we used serum‐starved HUVECs to generate EMPs (Supplementary Figure S3) to mimic the ischemic condition in ACS patients and compared these to the vehicle control form HUVECs cultured in normal conditions.^20^ We visualized by confocal microscopy that PKH26 red fluorescence‐labelled EMPs from HUVECs were incorporated into THP‐1 monocyte membranes following treatment for 2 and 4 hours, and internalized at 24 hours (Figure 1B), while PKH26 red fluorescent dye with the same procedures but without EMPs exhibited no significant incorporation and internalization, which ruled out the non‐specific lipophilic binding of non‐incorporated dye to the membrane of monocyte/macrophage (Supplementary Figure S4A). The monocytes treated with EMPs from dysfunctional ECs exhibited enhanced expression of pro‐inflammatory cytokines TNF‐α, IL‐1β, IL‐6, and IL‐8 (Figure 1C), as well as the protein expression of TNF‐α and IL‐1β (Figure 1D,E) compared to those treated with vehicle control from healthy HUVECs in normal culture conditions.

The NF‐кB pathway plays a crucial role in the initiation of inflammation.^26^ We further tested whether this signal pathway contributed to the enhanced pro‐inflammatory cytokine production when monocytes were treated with EMPs from dysfunctional ECs. Elevated expression of phosphorylated NF‐кB p65 subunit was observed in THP‐1 monocytes following the treatment with the EMPs for 4 hours (Figure 1F), which was confirmed by the observation under fluorescent microscopy that the EMPs promoted the nuclear translocation of NF‐кB p65 subunit after 4 hours, and returned to the cytoplasm after 8 hours (Figure 1G). Furthermore, we used the specific NF‐кB inhibitor PDTC (1 μM) and found that it inhibited the EMP‐mediated NF‐кB p65 subunit nuclear translocation (Figure 1H), and reversed the enhanced expression of phosphorylated NF‐кB p65 subunit (Figure 1F), and additionally normalized the EMP‐mediated elevation of pro‐inflammatory cytokine expression (IL‐1β, TNF‐α, IL‐6, or IL‐8) (Figure 1C), and the protein expression of TNF‐α and IL‐1β (Figure 1D,E).

In addition to the EMPs from serum‐starved HUVECs, the EMPs from HUVECs cultured under hypoxia conditions were studied. Similar incorporation into THP‐1 monocyte membrane (Supplementary Figure S4B), and elevated expression of phosphorylated NF‐кB p65 subunit with the PDTC reversal effects (Supplementary Figure S4C) were observed in THP‐1 monocytes when treated with the EMPs from HUVECs under hypoxia conditions. These results suggest that EMPs from dysfunctional ECs are incorporated into the monocyte membrane and enhance the expression of phosphorylated NF‐кB p65 subunit, which translocates to the nucleus and regulates the production of pro‐inflammatory cytokines TNF‐α and IL‐1β.

### 3.4 EMPs from dysfunctional ECs promote monocyte adhesion to ECs and macrophage proliferation

The important role of vascular macrophages for atherosclerosis progression and ACS aggravation can result from subendothelial infiltration from the blood or in situ proliferation of tissue‐resident macrophages, and cell adhesion and transmigration contribute to monocyte infiltration into the vessel wall.^27^, ^28^

**Figure 2:**
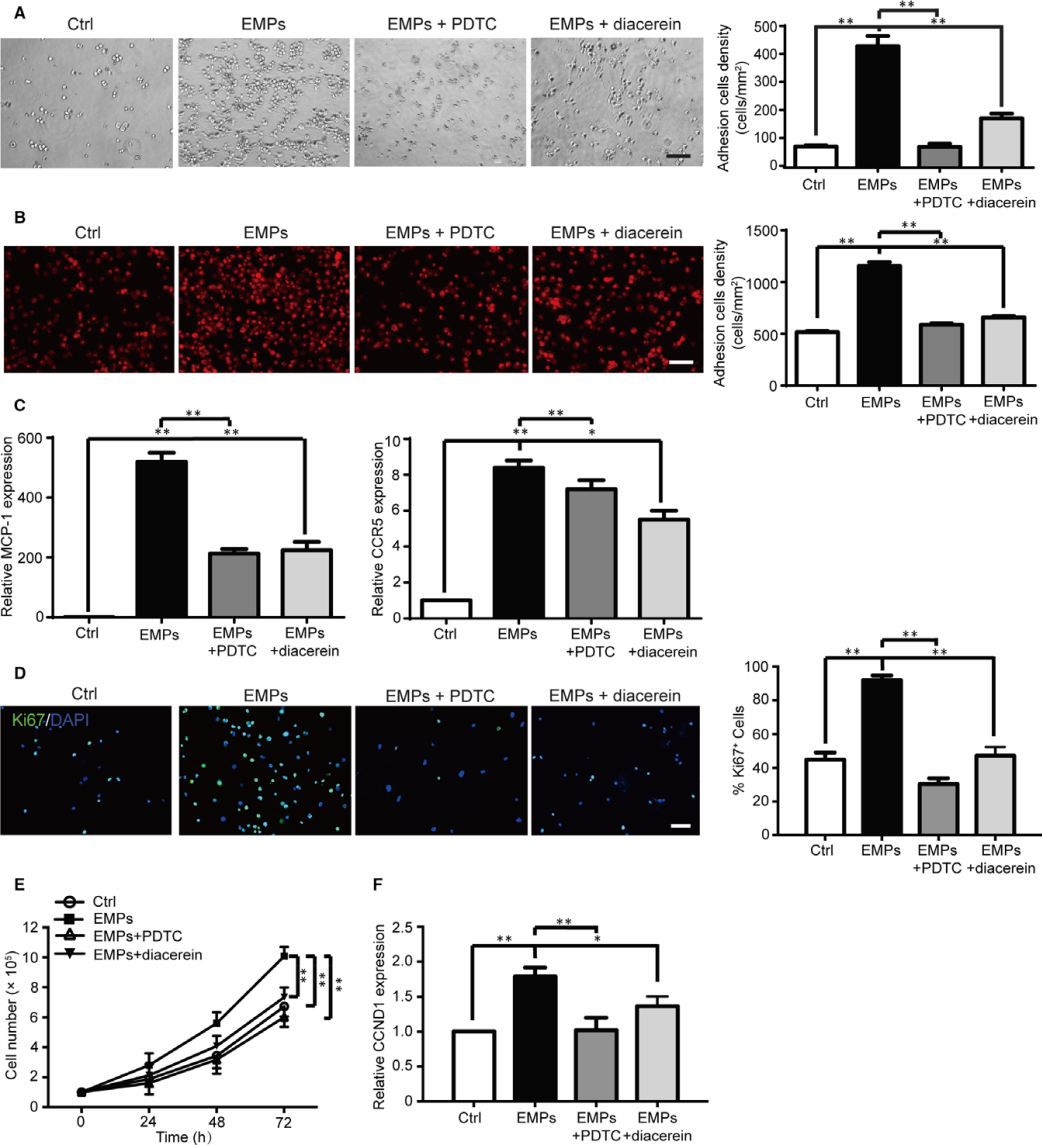
EMPs from dysfunctional ECs promote monocyte adhesion and proliferation. A‐B, THP‐1 monocyte adhesion to fibronectin‐coated surface (A) and adhesion of the PKH26 red fluorescence‐labelled THP‐1 monocyte to ECs (B) after incubation with vehicle control, EMPs from dysfunctional ECs, EMPs + PDTC (1 μM), and EMPs + diacerein (50 μM) for 48 hours with representative image shown in left panel and quantification in right panel. Scale bar: 100 μm. C, Quantitative PCR results show elevated expression of monocyte chemotactic genes MCP‐1 and CCR‐5 following treatment of the EMPs for 48 hours, which was reversed by NF‐кB specific inhibitor PDTC (1 μM) and IL‐1β specific inhibitor diacerein (50 μM). D, The representative image of immunostaining of Ki67 (Green), the proliferative marker, in THP‐1 monocytes following incubation vehicle control, EMPs from dysfunctional ECs, EMPs + PDTC (1 μM), and EMPs + diacerein (50 μM) for 24 hours in left panel, with quantification the percentage of Ki67 positive proliferating cells in right panel. Scale bar: 100 μm. E, Cell counting assay shows THP‐1 cell proliferation following treatment of vehicle control, EMPs from dysfunctional ECs and EMPs + PDTC (1 μM) or EMPs + diacerein (50 μM) for 24, 48, and 72 hours. EMPs from dysfunctional ECs promote cell proliferation which is reversed by PDTC (1 μM) and diacerein (50 μM). F, Quantitative PCR results show increased cyclin D1 (CCND1) expression following treatment of EMPs from dysfunctional ECs for 24 hours compared to those from ECs under normal culture condition, and PDTC (1 μM) and diacerein (50 μM) reversed the EMP‐induced elevate CCND1 expression. All data are presented as mean ± SEM (n = 5). *P < 0.05 and **P < 0.01

In this study, we found that the EMPs from dysfunctional ECs markedly increased the adhesion of monocyte/macrophage to the fibronectin‐coated surface (Figure 2A), as well as to HUVECs (Figure 2B). qPCR analysis showed that treatment with EMPs significantly elevated the expression of MCP‐1 and CC‐chemokine receptor‐5 (CCR‐5) in THP‐1 monocytes (Figure 2C). The NF‐кB specific inhibitor PDTC or IL‐1β specific inhibitor diacerein reversed the enhanced expression of MCP‐1 and CCR‐5, and the EMP‐mediated monocyte cell adhesion to ECs (Figure 2A‐C), suggesting that EMPs from dysfunctional ECs promote monocyte adhesion via increased MCP‐1 and CCR‐5 expression depending on the NF‐кB and IL‐1β pathways.

**Figure 3:**
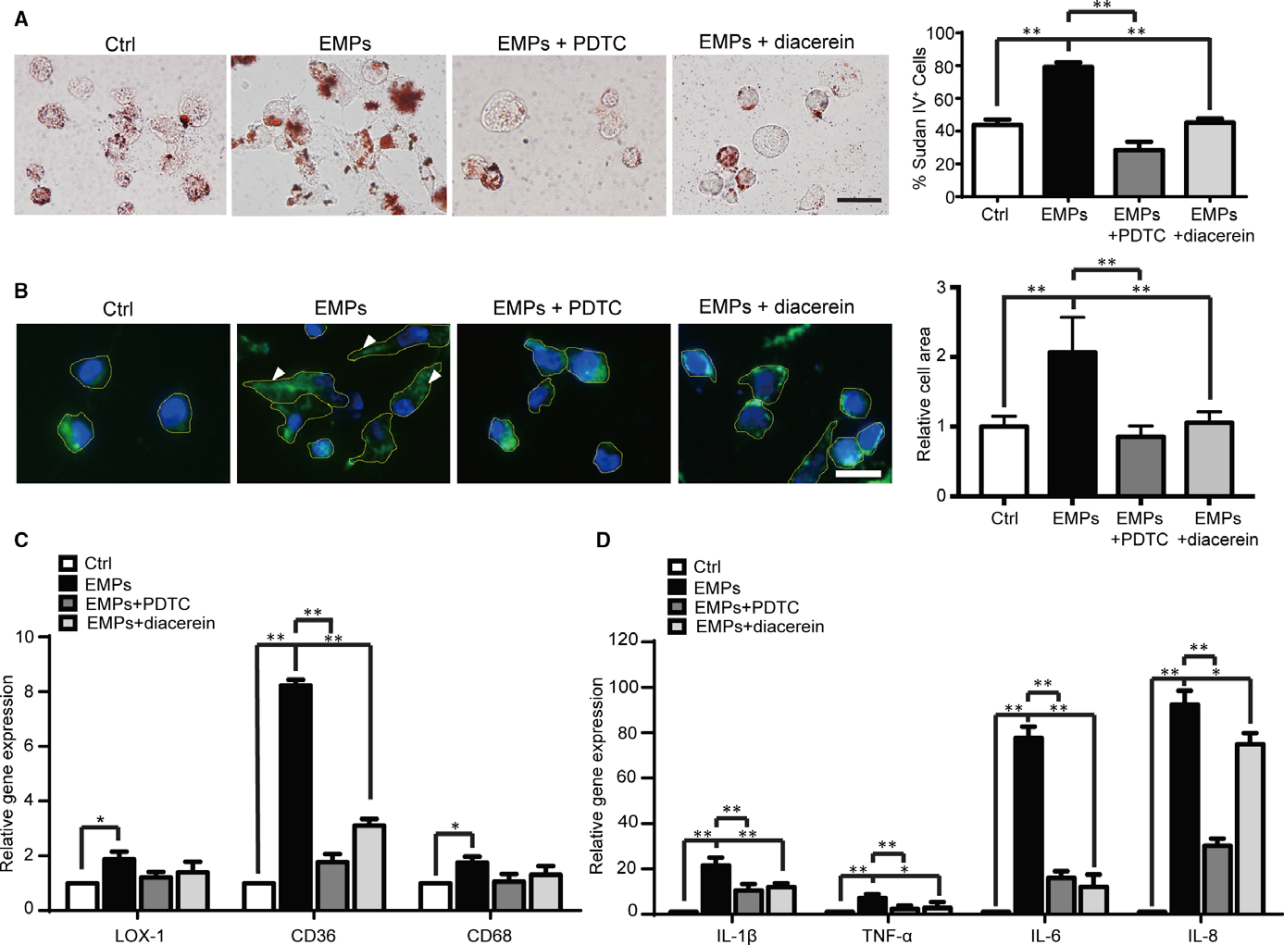
EMPs from dysfunctional ECs promote monocyte ox‐LDL phagocytosis and foam cell formation. A, The THP‐1 monocytes were incubated with vehicle control, EMPs from dysfunctional ECs, EMPs + PDTC, EMPs + diacerein for 48 hours, then ox‐LDL (50 μg/mL) was added to the THP‐1 cells for 9 hours. The representative image of ox‐LDL phagocytosis by THP‐1 cells as measured by Sudan IV staining of ox‐LDL uptake shown in left panel with quantification in right panel. Scale bar: 20 μm. B, The adherence of THP‐1 cells to a fibronectin surface was incubated with vehicle control, EMPs from dysfunctional ECs, EMPs + PDTC (1 μM), and EMPs + diacerein (50 μM) for 72 hours. The representative image of actin filaments staining by phalloidin (Green) in THP‐1 cells shown in left panel with arrow heads indicating cytoskeleton spreading, and quantification with relative cell area (circle) shown in right panel. Scale bar: 20 μm. C, Quantitative PCR results show elevated expression of phagocytic cell markers (CD36, CD68, and LOX‐1) in THP‐1 cells following treatment of EMPs from dysfunctional ECs compared to the vehicle control, and PDTC (1 μM) and diacerein (50 μM) reverse the EMP‐induced elevated expression of phagocytic cell markers. D, Quantitative PCR results show elevated expression of pro‐inflammatory cytokines in THP‐1 cells following treatment of EMPs from dysfunctional ECs compared to the vehicle control after further incubation with ox‐LDL for 9 hours. PDTC (1 μM) and diacerein (50 μM) reverse the EMPs treatment and ox‐LDL further incubation induced elevated expression of pro‐inflammatory cytokines. All data are presented as mean ± SEM (n = 5), *P < 0.05 and **P < 0.01

The tissue‐resident macrophage proliferation also contributes to the increased macrophages in the vessel wall. Immunostaining with Ki67 and cell count assay were used to evaluate cell proliferation, and the results showed that EMPs from dysfunctional ECs stimulated macrophage proliferation (Figure 2D,E). qPCR analysis showed elevated expression of Cyclin D1 (CCND1), a gene for regulation of cell cycle G1/S transition (Figure 3F). Further studies indicated that the PDTC and diacerein reversed the increased CCND1 expression and normalized the EMP‐mediated enhanced cell proliferation (Figure 2D‐F), suggesting that EMPs from dysfunctional ECs promote macrophage proliferation via regulation of CCND1 through the NF‐кB and IL‐1β signal pathways.

These results suggest that EMPs from dysfunctional ECs can promote monocyte adhesion via NF‐кB and IL‐1β‐dependent enhanced expression of MCP‐1 and CCR‐5, while promoting macrophage proliferation via NF‐кB and IL‐1β‐dependent enhanced CCND1 expression.

### 3.5 EMPs promote monocyte ox‐LDL phagocytosis and foam cell formation

Not only are the numbers of monocytes/macrophages important, the cell functional status also contributes to the inflammation of the vessel wall. The formation of foam cells from infiltrated monocytes/macrophages in the vessel wall occurs when phagocytosis of ox‐LDL produces pro‐inflammatory cytokines, which is a critical component in progression of atherosclerotic plaque development leading to the deterioration of ACS or even occurrence of MI.^1^ Therefore, we tested whether the EMPs from dysfunctional ECs promoted ox‐LDL phagocytosis by monocytes/macrophages and formation of foam cells.

The results demonstrated that more ox‐LDL engulfing foam cells were formed following treatment of THP‐1 monocytes with EMPs from dysfunctional ECs (Figure 3A). Actin filament cytoskeletal remodelling, including formation of filopods and lamellipods, is required for the process of phagocytosis of monocytes/macrophages and macrophages with filopods and lamellipods upon activation have higher phagocytic capability.^29^, ^30^ Immunostaining of actin filaments by Alexa Fluor 488 conjugated‐ phalloidin was performed and quantification of actin filament cytoskeletal spreading shown by relative cell area. The results showed that the EMPs from dysfunctional ECs promoted cytoskeleton spreading in monocyte/macrophages (Figure 3B) and these high‐capacity phagocytic cells exhibited elevated expression levels of phagocytic cell markers (LOX‐1, CD36, and CD68) (Figure 3C), and produced more pro‐inflammatory cytokines IL‐1β, TNF‐α, IL‐6, and IL‐8 (Figure 3D).

NF‐κB is a ubiquitous transcription factor which controls the expression of various genes including those involved in lamellipod formation and phagocytosis.^31^, ^32^ This study showed that PDTC and diacerein reversed the EMP‐mediated increased cytoskeletal spreading and elevated ox‐LDL phagocytosis, confirming that the processes are dependent on the NF‐κB and IL‐1β pathways.

These results suggest that EMPs from dysfunctional ECs can increase monocyte surface spreading and promote monocyte phagocytic activity depending on the NF‐κB and IL‐1β signal pathways to form foam cells, which further enhance inflammatory cytokine production and increase vascular inflammation.

## 4 DISCUSSION

The endothelium is a vital organ whose homeostasis is essential for normal vascular physiology, and endothelial dysfunction has been proved to be a critical factor in the initiation and progression of atherosclerosis.^1^, ^2^ Endothelial cell activation and apoptosis, both key elements in the development of vascular disease, lead to EMPs release. Accumulating evidences show that circulating EMP levels are significantly increased in conditions associated with endothelial dysfunction, such as coronary artery diseases and ACS.^6^, ^7^

EMPs are endothelial plasma membrane blebbings ranging in size between 0.1 and 1 μm and carrying endothelial proteins such as vascular endothelial cadherin, PECAM, ICAM‐1 and E‐selectin, and apoptotic markers. In this study, we used flow cytometry with FITC‐labelled anti‐Annexin V (apoptotic marker) and PE‐labelled anti‐CD144 (endothelial marker) to identify and quantify EMPs and the result confirmed increased circulatory EMP levels in patients with ACS who also tended to have higher serum levels of hs‐CRP, as well as increased TNF‐α and IL‐1β in the plasma.

Recent studies found that EMPs can affect inflammation, blood coagulation, and vascular functions and are closely related to the occurrence of cardiovascular disease.^15^, ^16^, ^33^ EMPs released from endothelial cells carry a variety of biologically active substances, which mediate inflammatory responses and cell signal transduction and substantially elevate a variety of inflammatory cytokines by which EMPs may contribute to cardiovascular disease progression.^7^, ^18^ Therefore, we hypothesize that EMPs might be involved in the regulation of monocyte/macrophage function for the production of pro‐inflammatory cytokines.

Flow cytometry can assess EMPs in patients’ plasma, but unfortunately it is technically difficult to separate EMPs from patients for further functional evaluation. Recently one study collected EMPs from the supernatant of apoptotic human coronary arterial endothelial cells (HCAECs) after starvation for functional study of EMPs.^20^ Patients with ACS have temporary myocardial ischaemia as shown in this study by significantly increased hs‐TnT in ACS patients compared to the non‐ACS patients, therefore HUVECs cultured with starvation and under hypoxic condition 20, 22 were used to generate EMPs, which might mimic the dysfunctional ECs in ACS patients. The results demonstrated that the EMPs derived from HUVECs were incorporate into the macrophage membrane and promoted the NF‐кB p65 subunit phosphorylation and translocation to the nucleus to increase the production of pro‐inflammatory cytokines.

Dysfunctional ECs promote leukocyte adhesion and migration, which is a critical component in the initiation and progression of atherosclerotic lesion development.^1^, ^2^ Patients with MI had higher VCAM‐1 expression on coronary artery ECs which would promote monocyte adhesion and infiltration into the vessel wall.^34^ Our study showed that the EMPs upregulated the expression of MCP‐1 and CCR‐5, the well‐known signals which promote monocyte adhesion to ECs, was dependent on the IL‐1β and NF‐кB signal pathways.

The NF‐кB transcription factor family is a pleiotropic regulator of many cellular pathways, providing a mechanism for the cell to respond to a wide variety of stimuli and environmental challenges.^35^ The regulation by NF‐кB on the cell cycle was first described in 1991, when NF‐кB DNA binding was found to increase during G0/G1 transition in mouse fibroblasts, and the cyclin D1 encoding gene CCND1 is the most well‐established cell cycle regulator that is an NF‐кB target gene with its promoter containing three NF‐кB binding sites. Our study indicated that the EMPs from dysfunctional ECs promoted monocyte proliferation as measured by Ki67 staining and cell count assay, as well as increased the CCND1 expression, and PDTC, the NF‐кB specific inhibitor, and diacerein, the IL‐1β inhibitor reversed the upregulation of CCND1, and further normalized the enhanced monocyte proliferation, suggesting EMPs derived from dysfunctional ECs promote monocyte proliferation via the NF‐кB and IL‐1β‐mediated CCND1 signal pathways.

Beyond their roles in monocyte adhesion and proliferation to increase the inflammatory cell number in the blood vessels, EMPs also influence macrophage functional status which contributes to vascular inflammation and atherosclerotic lesion development. Our study demonstrated that EMPs were incorporated into the monocyte membrane and promoted the NF‐кB p65 subunit phosphorylation and further translocation to the nucleus to increase the production of pro‐inflammatory cytokines.

The phagocytosis of ox‐LDL by macrophages and the subsequent formation of foam cells are crucial for atherosclerotic lesion development.^1^, ^2^ Cell surface spreading, including formation of filopods and lamellipods, is required for macrophage phagocytosis.^29^, ^30^ Ox‐LDL phagocytosis by macrophages occurs mainly through scavenger receptors, among which CD36 is considered to be the most important.^36^ The present study showed that EMP‐treated monocytes/macrophages exhibited increased cytoskeleton spreading with more capability to engulf ox‐LDL to form foam cells, which showed elevated phagocytic cell markers such as CD36, LOX‐1, and CD68, and these foam cells further secrete even more inflammatory cytokines and increase vascular inflammation.

This study has limitation including relative small sample size and usage of EMPs from dysfunctional endothelial cell line. It would be ideal to use EMPs from ACS and non‐ACS patients to assess the different functions ex vivo. However, there may also be many other interacting factors in ACS patients, such as different background cardiovascular risk factors and differences in treatments used that may complicate such an analysis. It was also not practical to separate EMPs from patients’ samples that had been collected, the patient plasma has many MPs from different origins that will not reflect the function of EMPs, and more reproducible EMPs from endothelial cell line was adopted for further functional study.

In summary, ACS patients have higher circulatory EMP levels, which may participate in the regulation of NF‐кB and IL‐1β signal pathways of monocytes/macrophages and influence cell adhesion, proliferation, and macrophage phagocytic capability, contributing to the vascular inflammation associated with atherosclerosis progression in ACS patients. Therefore, future targeting of EMP‐mediated inflammatory responses in vessel wall may be a promising therapeutic strategy to limit the progression of atherosclerotic lesion formation post‐ACS.

## CONFLICT OF INTEREST

The authors declare that they have no conflicts of interest.

## AUTHOR CONTRIBUTIONS

YF W, J L, XL C, and S P conceived and designed the study, together HM S, YS K, JJ P, and T Z performed the experiments; L Z, ZR Y, B T, P C, and YH C guided the study; YZ Z and Y L designed the study and wrote the manuscript.
