# Supplementary material for: Dysfunctional endothelial‐derived microparticles promote inflammatory macrophage formation via NF‐кB and IL‐1β signal pathways
Source: J Cell Mol Med. 2018 Oct 18;23(1):476–86. doi: 10.1111/jcmm.13950 (PMC6307808; doi:10.1111/jcmm.13950)

Supplementary Material

Supplementary Table 1: Primer sequence for real time quantitative PCR (RT-qPCR)

Gene Name	Primer Sequence	
IL-1β	Forward	5'-ACAGTGGCAATGAGGATG-3‘
	Reverse	5’-TGTAGTGGTGGTCGGAGA-3‘
TNF-α	Forward	5’-ACTTTGGAGTGATCGGCCC-3‘
	Reverse	5’-CATTGGCCAGGAGGGCATT-3‘
IL-6	Forward	5’-ACTCACCTCTTCAGAACGAATTG-3‘
	Reverse	5’-CCATCTTTGGAAGGTTCAGGTTG-3‘
IL-8	Forward	5’-TTTTGCCAAGGAGTGCTAAAGA-3'
	Reverse	5'-AACCCTCTGCACCCAGTTTTC-3'
CCND1	Forward	5'-CAATGACCCCGCACGATTTC-3'
	Reverse	5'-CATGGAGGGCGGATTGGAA-3'
MCP-1	Forward	5'-CAGCCAGATGCAATCAATGCC-3'
	Reverse	5'-TGGAATCCTGAACCCACTTCT-3'
CCR5	Forward	5'-TGATTTGCACAGCTCATCTGG-3'
	Reverse	5'-GGCTGCGATTTGCTTCACAT-3'
LOX-1	Forward	5’-ACTCTAGGGGTCCTTTGCCT-3’
	Reverse	5’-TAGGAGGTCAGACACCTGGG-3’
CD36	Forward	5’-AAGCCAGGTATTGCAGTTCTTT-3’
	Reverse	5’-GCATTTGCTGATGTCTAGCACA-3’
CD68	Forward	5’-TGGGGCAGAGCTTCAGTTG-3’
	Reverse	5’-TGGGGCAGGAGAAACTTTGC-3’
GAPDH	Forward	5'-ATGGAAATCCCATCACCATCTT-3'
	Reverse	5'-CGCCCCACTTGATTTTGG-3'

Supplementary figures:

Figure. S1: Flow cytometry analysis of circulating EMPs in ACS and non-ACS patients. (A) Representative image of flow cytometry analysis of EMPs in non-ACS patients. (B) Representative image of flow cytometry of EMPs in ACS patients. The left image is log forward scatter/log side scatter dot plot and the square indicating the MP population less than 1 μm calibrant beads, the right image is fluorescent analysis of FITC-labeled anti-Annexin V and PE-labeled anti-CD144.

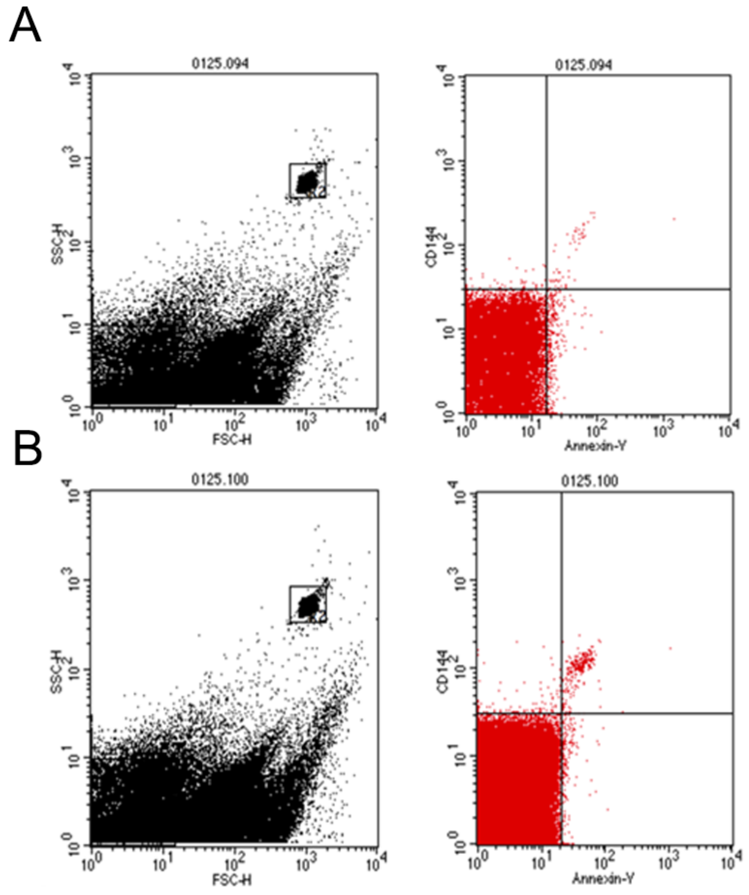

Figure. S2: Heat map of plasma protein array of ACS or non-ACS patients. The results showed increased protein expression of TNF-α, IL-1β and MCP-1 in ACS patients compared to non-ACS patients.

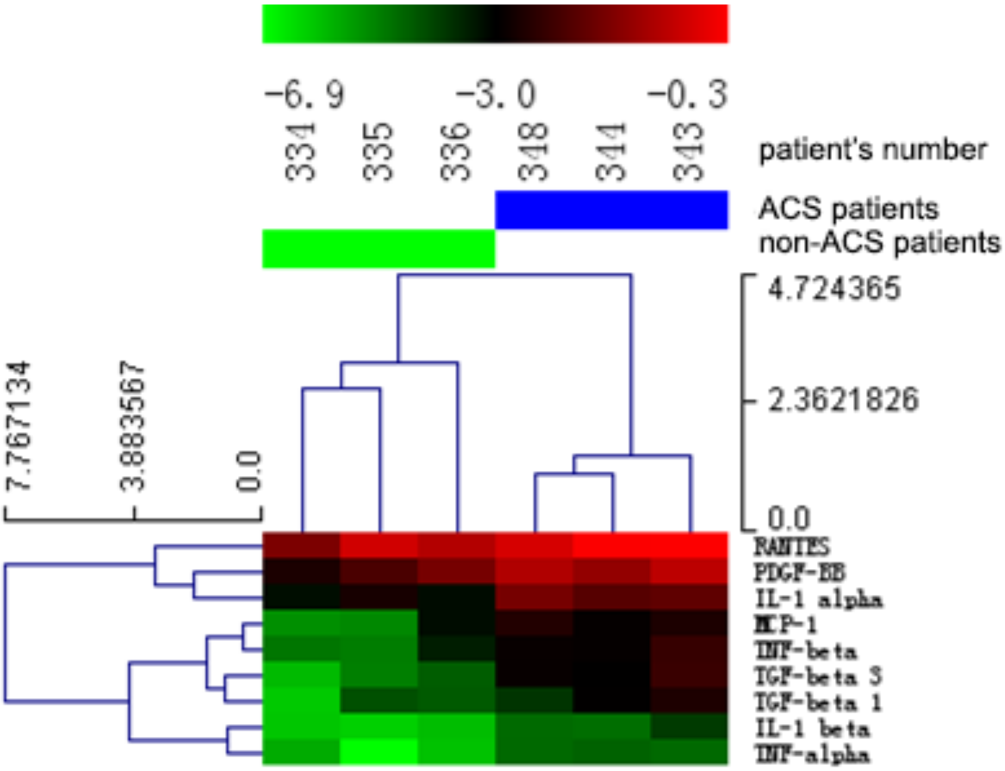

Figure. S3: Flow cytometry analysis of EMPs from starved HUVECs. The left image is log forward scatter/log side scatter dot plot and the square indicating the MP population less than 1 μm calibrant beads, the right image is fluorescent analysis of FITC-labeled anti-Annexin V and PE-labeled anti-CD144.

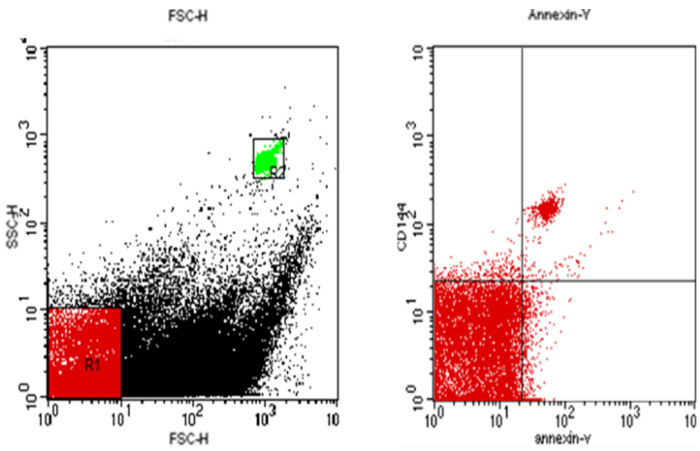

Figure. S4: EMPs derived from hypoxic HUVECs are incorporated into the membrane of THP-1 monocyte and activate NF-κB p65 signal. (A) The same amount of PKH26 red fluorescent dye without EMP in the medium was used as a control for dye-labeled EMPs derived from HUVECs and the results showed no significant non-specific lipophilic binding of the dye to the membrane of THP-1 monocytes. (B) The EMPs collected from HUVECs under hypoxia condition (2% O_2, 5% CO_2 balanced with N_2) for 24 hours are labeled with PKH26 red fluorescence and added to the THP-1 culture medium. The EMPs are incorporated into the membrane of THP-1 monocyte membrane visualized under a confocal microscopy at 2 hours. Scale bar: 10 μm. (C) Western blot shows increased NF-κB (p65 subunit) phosphorylation of THP-1 cells after 4 hours incubation with EMPs from HUVECs under hypoxia culture condition, which is reversed by PDTC (1 μM).

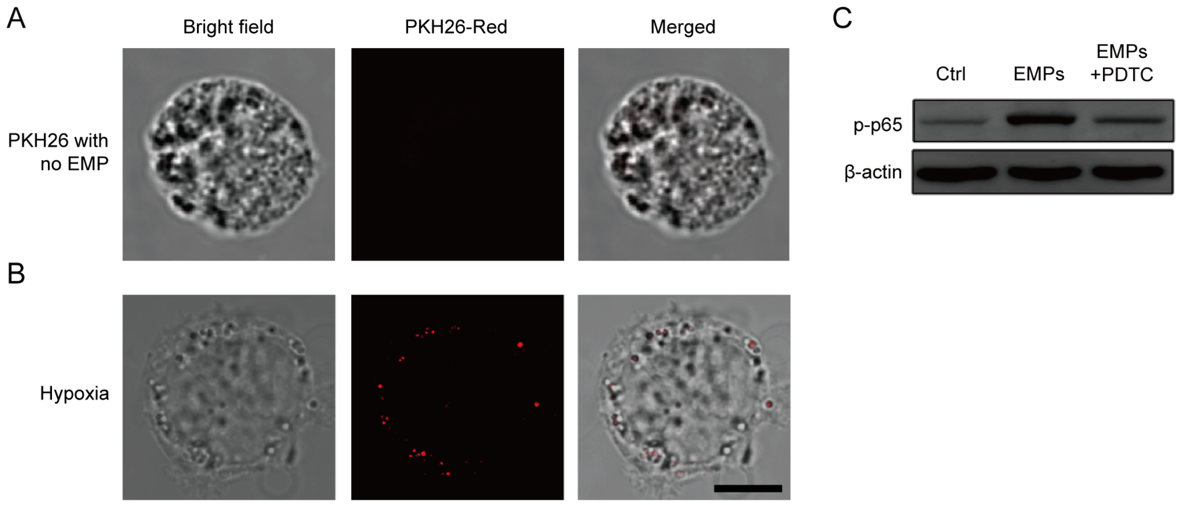

Supplement: Supplementary file 1 [file JCMM-23-476-s001.pdf]
